# Ultrasound-assisted tannic acid crosslinking of myofibrillar protein-stabilized high internal phase emulsions: Enhanced stability, mechanical properties, and 3D printability

**DOI:** 10.1016/j.ultsonch.2025.107703

**Published:** 2025-12-02

**Authors:** Zitong Dong, Feiyu Zhang, Peng Wang, Xinglian Xu

**Affiliations:** State Key Lab of Meat Quality Control and Cultured Meat Development, Ministry of Science and Technology, Key Laboratory of Meat Processing, Ministry of Agriculture, College of Food Science and Technology, Nanjing Agricultural University, Nanjing, Jiangsu 210095, China

**Keywords:** Myofibrillar protein, Tannic acid, High-intensity ultrasound, High internal phase emulsions, Interfacial rheology

## Abstract

The study investigated the synergistic effects of high ultrasound (HS) treatment and tannic acid (TA) conjugation on the structural and interfacial properties of myofibrillar protein (MP), and their roles in stabilizing high internal phase emulsions (HIPEs). Spectroscopic and interfacial analyses showed that HS promoted TA-induced covalent conjugation via exposure of reactive groups, driving conformational rearrangements and formation of more compact aggregates. At moderate TA concentrations, smaller particle size and enhanced interfacial performance were observed for MP–TA conjugates, as evidenced by accelerated adsorption kinetics and elevated dilatational moduli of interfacial films. In HIPEs, these changes yielded smaller and more uniform droplets, higher viscosity, larger linear viscoelastic regions, and greater yield stress. These improvements resulted in greater resistance to coalescence and oiling-off under storage, heating, and centrifugal stress. MP-based HIPEs with 10μM g^−1^ TA and 240 W HS exhibited the most favorable balance of droplet architecture, rheology and stability. In addition, the optimized HIPEs displayed good printability in a preliminary 3D printing test, highlighting their potential for structuring food matrices. Overall, this work elucidates how HS-assisted TA conjugation alters MP conformation, interfacial assembly and emulsion rheology, providing a mechanistic basis for designing robust MP-stabilized HIPEs.

## Graphical abstract

**Figure:**
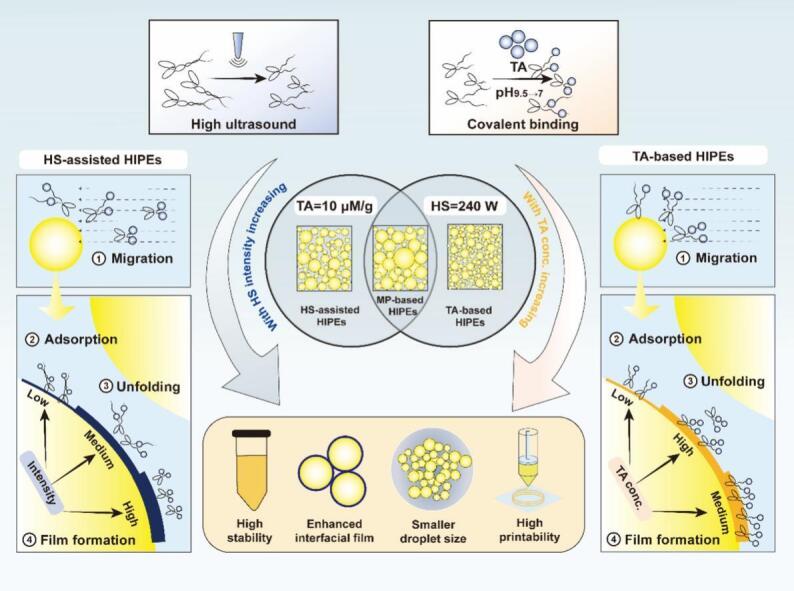

## 1 Introduction

High internal phase emulsions (HIPEs), defined as emulsions with internal phase fractions above 74 %, exhibit densely packed droplets and solid-like textures, making them attractive for fat replacement, bioactive encapsulation, and novel food structuring [1]. In response to the demand for clean-label food systems, protein-based HIPEs have recently attracted attention. Among these, myofibrillar protein (MP) stands out due to its abundance, balanced amino acid profile, high bioavailability, and nutritional value, as well as its general recognition as a low-allergenic and clean-label ingredient [2]. However, MP-stabilized HIPEs often exhibit limited interfacial elasticity and rigidity, leading to droplet coalescence and deformation under thermal or mechanical stress, highlighting the need to enhance MP functionality, particularly interfacial assembly [3,4].

Given these limitations, polyphenols have been widely explored as protein modifiers owing to their multiple hydroxyl groups and strong redox activity [5]. Conjugation with polyphenols has been shown to alter various protein properties: for instance, whey protein coupled with epigallocatechin gallate (EGCG) exhibited higher oxidative stability and interfacial adsorption [6], while fish myofibrillar protein combined with tea polyphenols displayed greater emulsifying activity and thermal stability [7]. These findings indicate that polyphenols can effectively modulate protein structures and performance. However, many of these improvements were achieved mainly through noncovalent interactions such as hydrogen bonding and hydrophobic association [8]. Although such interactions may enhance the protein solubility and surface activity, the complexes are reversible and sensitive to environmental conditions, which limits their stability during processing. In contrast, covalent conjugation creates irreversible linkages between polyphenols and protein side chains, stabilizing protein structures and improving resistance to denaturation and aggregation [9,10]. Thus, although previous work has highlighted the promise of protein–polyphenol systems, there is still limited information on how covalent modification specifically influences MP interfacial behavior and HIPE stability.

Among polyphenols capable of covalent modification, tannic acid (TA) is particularly attractive due to its abundant galloyl groups, strong antioxidant activity, and recognized food-grade safety [11]. TA forms multiple covalent linkages with protein residues, providing a more robust and stable modification compared with other polyphenols such as EGCG or chlorogenic acid [12]. Despite these advantages, the structural consequences of MP-TA conjugation in HIPE systems are still not fully characterized.

Beyond chemical conjugation, high-intensity ultrasound (HS) treatment has also been used to enhance protein functionality. HS produces acoustic cavitation, generating localized shear forces and microjets that can disrupt protein aggregates, unfold molecules, and expose buried reactive groups [13]. These changes improve protein solubility and interfacial activity and facilitate subsequent covalent conjugation with polyphenols [14]. Previous studies suggest that HS-assisted conjugation accelerates reaction kinetics and improves protein functionality [15]. Despite these interesting reports, relatively few studies have directly focused on how HS-assisted conjugation with polyphenols regulates MP structure and interfacial properties.

The study aimed to examine the effects of TA conjugation under HS on MP structural characteristics, interfacial adsorption, dilatational rheology, and to link these changes with the microstructure, rheology, and stability of HIPEs. Besides, this work establishes multi-scale relationships from protein conformation to macroscopic performance by combining spectroscopic analysis, interfacial rheology, and emulsion characterization. Based on this framework, we hypothesized that ultrasound-assisted TA conjugation would strengthen MP–TA covalent interactions and induce pronounced conformational rearrangements, which would subsequently enhance MP interfacial adsorption and increase the dilatational elasticity of the interfacial film, ultimately yielding HIPEs with improved structural stability, rheological performance, and 3D printing fidelity when TA concentration and HS intensity are appropriately balanced. These findings provide mechanistic insights into how HS-assisted TA conjugation regulates MP structure and interfacial behavior, and establish a mechanistic basis for the rational design of robust MP-stabilized HIPEs in food structuring.

## 2 Materials and methods

### 2.1 Materials

Chicken breast meat: Fresh chicken breast meat was obtained from Sushi Meat Co., Ltd. (Nanjing, Jiangsu, China). All samples were transported and stored under a cold chain at 4 °C.

Tannic acid (purity ≥ 98 %) was purchased from Macklin Reagent Co. Ltd. (Shanghai, China). Potassium bromide (KBr, spectral grade, purity ≥ 99.9 %) was used for Fourier-transform infrared (FTIR) spectroscopy analysis. Analytical grade chemicals, including potassium chloride (KCl), potassium dihydrogen phosphate (KH_2_PO_4_), and dipotassium hydrogen phosphate (K_2_HPO_4_) (purity ≥ 99 %), were obtained from Sinopharm Chemical Reagent Co., Ltd. (China).

### 2.2 Extraction of MP

MP was extracted by our previous method [16]. Briefly, trimmed meat was minced and mixed with pre-chilled extraction buffer (0.1 M KCl, 20 mM K_2_HPO_4_/KH_2_PO_4_, 2 mM MgCl_2_, 1 mM EGTA, pH 7.0, 4 °C) at a ratio of 1:4 (w/v) and was homogenized twice at 6,900 rpm for 30 s under ice-bath conditions. The homogenate was filtered, centrifuged (2,000 × g, 10 min, 4 °C) and the precipitate washed three times with the same buffer.

Subsequently, the precipitate was resuspended in four volumes of pre-chilled 0.1 M KCl solution, centrifuged (2,500 × g, 10 min, 4 °C) and washed twice. The MP was stored at 4 °C, and its concentration was determined using the Bradford assay, with bovine serum albumin (BSA) as the standard.

### 2.3 Preparation of MP-TA conjugates

**Fig. 1:**
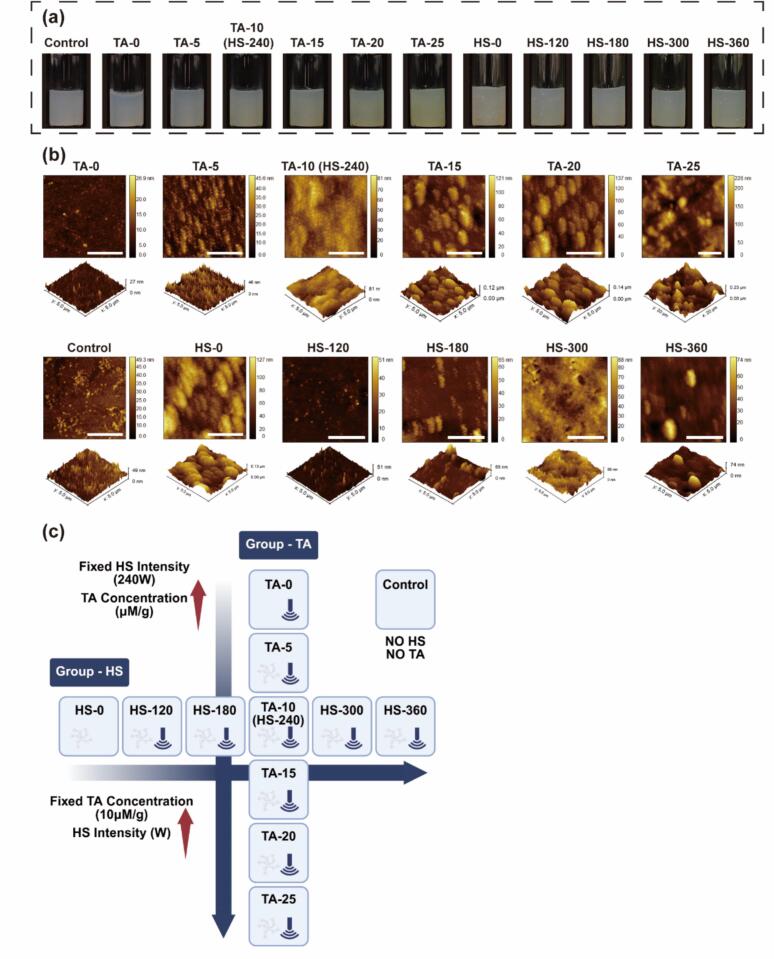
Visual appearance (a) and AFM images (b) of MP and different MP-TA conjugates. The experimental design (c). HS intensity variation group (Group-TA): TA concentration was fixed at 10 μM/g protein, and HS intensity was varied across a gradient (0, 120, 180, 240, 300 and 360 W). TA concentration variation group (Group-HS): HS intensity was fixed at 240 W, and TA concentrations were varied across a gradient (0, 5, 10, 15, 20 and 25 μM/g protein).

To systematically investigate the synergistic modification of MP by high-intensity ultrasound (HS) and TA, two single-variable experimental designs were adopted: HS intensity variation group (Group-TA): TA concentration was fixed at 10 μM/g protein, and HS intensity was varied at 0, 120, 180, 240, 300, and 360 W. TA concentration variation group (Group-HS): HS intensity was fixed at 240 W, and TA concentrations were varied at 0, 5, 10, 15, 20, and 25 μM/g protein. Details of the experimental design are provided in Fig. 1c. These parameter levels were selected based on preliminary rheological screening (data not shown), which showed that HIPEs prepared at 10 μM/g TA and 240 W HS exhibited intermediate mechanical strength. The preparation procedure for the conjugates was as follows: TA powder was directly added to the MP solutions (20 mg/mL, dissolved in phosphate-buffered saline (PBS) containing 0.6 M KCl, pH 7.0). Immediately thereafter, the solution was subjected to pulsed ultrasound treatment using a 20-kHz ultrasonic processor (YM-1500Y, Shanghai Yuming Instrument Co., Ltd., China). The probe (1  cm in diameter) was inserted 1  cm below the liquid surface, and ultrasound was applied at the designated power level for 10 min in a pulsed mode (2 s on/3 s off), with continuous temperature control via ice bath. Finally, the pH was readjusted to 7.0 using 0.2 M HCl. The resulting MP-TA conjugates were stored at 4 °C for the following tests.

### 2.4 Characterization of MP-TA conjugates

#### 2.4.1 Atomic force microscopy (AFM)

The microstructures of samples under different treatments were observed by AFM (Dimension ICON, Bruker Corp., USA). Specifically, sample solutions (2 mg/mL) were deposited onto freshly cleaved mica and air-dried at room temperature (25 ± 1 °C) for 2 h. Imaging was performed in tapping mode over a 5 μm × 5 μm area at a scan rate of 1.0 Hz. AFM images were processed using the Gwyddion software, and a second-order plane fitting algorithm was applied to correct for substrate tilt. To ensure representative results, at least three randomly regions were scanned for each sample.

#### 2.4.2 Particle size and zeta potential

The hydrodynamic diameter and zeta potential of the samples were measured using dynamic light scattering (DLS) with a Zetasizer Nano ZS 90 instrument (Malvern Instruments, UK), following the method of Tao et al. [17]. MP and MP-TA conjugates were first diluted to 0.5 mg/mL using PBS buffer (0.6 M KCl, pH 7.0), and 1.3 mL of the solution was transferred to a quartz cuvette. After equilibration at 25 ± 0.1 °C for 120 s, particle size was determined.

For zeta potential measurements, samples were diluted to 0.1 mg/mL with ultrapure water, and 750  μL was transferred to a DTS1070 folded capillary cell. Prior to each measurement, both the cuvette and the capillary cell were rinsed three times with anhydrous ethanol, then with ultrapure water, and finally with the sample solution. Each sample was measured in triplicate to ensure reproducibility.

#### 2.4.3 Shear viscosity

The shear viscosity of protein solutions was measured using a rotational rheometer (Physica MCR 302e, Anton Paar, Austria) equipped with a 50 mm diameter parallel plate and a 1 mm gap. Measurements were conducted at 25 °C with a shear rate range from 0.1 s^−1^ to 1000 s^−1^, and apparent viscosity was recorded accordingly. Each sample was measured in triplicate to ensure reproducibility.

#### 2.4.4 Fourier transform infrared spectroscopy (FTIR)

The structural characteristics of samples were analyzed by FTIR (Nicolet iS10, Thermo Scientific, USA). Firstly, MP and MP-TA conjugates were frozen at − 80 °C for 24 h and subsequently lyophilized in a freeze dryer (CHRIST, Germany) for 36 h. The dried powders were mixed with pre-dried KBr at a ratio of 1:50 (w/w), ground in an agate mortar, and pressed into transparent discs (∼0.5 mm thick) under 10 MPa. Spectra were collected in transmission mode over the range of 4000–400 cm^−1^ with 64 scans at a resolution of 4 cm^−1^. For each sample, three parallel discs were prepared, and background correction was performed using KBr. Spectral processing was conducted using OMNIC 9.2 software, including automatic baseline correction and atmospheric compensation.

Secondary structure composition was analyzed by Gaussian curve fitting of the amide I region (1600–1700 cm^−1^) using Peak Fit v4.12 software. Characteristic peak assignments were as follows: α-helix (1650–1660 cm^−1^), β-sheet (1610–1640 cm^−1^), random coil (1640–1650 cm^−1^), and β-turn (1670–1690 cm^−1^) [18]. Baseline correction and deconvolution were applied before fitting. Relative contents were calculated from the integrated area of sub-peaks with R^2^ > 0.995. Each sample was analyzed in triplicate, and results were expressed as mean ± standard deviation.

#### 2.4.5 Interfacial adsorption kinetics

The interfacial adsorption behavior of sole MP and MP-TA conjugates at the oil–water interface was evaluated using a pendant drop tensiometer (Tracker, Teclis, France). Soybean oil was purified following the method of Zhang et al. [19], involving silica gel column chromatography and molecular sieve filtration to remove oxidation products and polar impurities. During measurement, 25 mL of protein solution (0.1 mg/mL) was added to a quartz sample cell. A glass syringe (500  μL, SGE, Australia) filled with purified oil was inserted into the protein solution. A 4  μL pendant oil droplet (axial ratio: 0.85–0.90) was formed at the tip of a U-shaped needle (0.84  mm outer diameter) using a computer-controlled stepper motor. Droplet shape evolution was monitored in real time using a high-speed CCD camera (200 fps) for 10,800 s. Dynamic interfacial tension (mN/m) was calculated using the Young–Laplace equation. All measurements were performed in triplicate.

#### 2.4.6 Interfacial dilatational rheology

Interfacial rheological properties were determined at room temperature using a slightly modified method based on Zhang et al. [19]. Dilatational behavior was assessed using the pendant drop method (Tracker, Teclis, France) as described in Section 2.4.5. A 4  μL droplet of purified soybean oil was immersed in 25 mL of protein solution (0.1 mg/mL), and a sinusoidal area strain of 20 % (ΔA/A) was applied at a fixed frequency of 0.05 Hz (20 s period). The test was conducted in intermittent mode, with five oscillation cycles followed by five relaxation cycles. Data were continuously recorded for 10,800 s. A Fourier transform was used to analyze the stress–strain response, and the interfacial dilatational modulus E (mN/m), storage modulus E_d_ (mN/m), and loss modulus E_v_ (mN/m) were calculated using the following equations:

(1) $$\sigma =\sigma_{0}\sin\left(\omega \theta +\delta \right)$$

(2) $$A=A_{0}\sin\left(\omega \theta \right)$$

(3) $$E=\frac{d\sigma }{\mathrm{dA}/A}=\frac{d\pi }{\mathrm{dlnA}}$$

(4) $$E_{d}=\left|E\right|cos\delta$$

(5) $$E_{v}=\left|E\right|sin\delta$$

where σ and σ_0_ were the dilatational stress at times of θ and 0 min, δ was the phase angle between stress and strain, A and A_0_ were the surface area of oil drop at times of θ and 0 min. Each sample was tested in triplicate at minimum.

#### 2.4.7 Intrinsic fluorescence spectroscopy

Intrinsic fluorescence spectra of protein samples were recorded using a multifunctional microplate reader (Spark, America). Protein solutions were diluted to 1 mg/mL with 0.1 M PBS (pH 7.0), and 200 μL aliquots were transferred into black 96-well microplates. The excitation wavelength was set at 275 nm (bandwidth: 20 nm), and emission spectra were recorded from 320 to 400 nm at 5 nm intervals with a scanning speed of 100 nm/min. Each sample was analyzed in triplicate.

#### 2.4.8 Reactive sulfhydryl content

Reactive sulfhydryl (R-SH) content was determined by Ellman’s method. Briefly, 800 μL of Tris-HCl buffer (0.1 M, pH 8.0) was mixed with 100 μL of protein solution (20 mg/mL, 0.6 M PBS, pH 7.0). For the blank control, the protein solution was replaced with an equal volume of PBS. Next, 100 μL of freshly prepared 1 mM DTNB (5,5′-dithio-bis (2-nitrobenzoic acid)) solution—prepared by dissolving 39.6 mg of DTNB in 100 mL of Tris-HCl buffer—was added. After thorough vortexing, the mixture was incubated at 25 ± 1 °C in the dark for 30 min. Absorbance was measured at 412 nm using a multifunctional microplate reader. The reactive sulfhydryl content (R-SH, nmol/mg) was calculated using the following equation (6):

(6) $$R-SH\left(nmol/mg\right)=\frac{A^{412}}{C}\times 73.53\times D$$

where A_412_, C and D were the absorbance, protein concentration and dilution factor, respectively.

#### 2.4.9 Surface hydrophobicity

Surface hydrophobicity was determined using the 8-anilino-1-naphthalenesulfonic acid (ANS) fluorescence method. MP-TA conjugates with MP concentrations of 0.1, 0.2, 0.3, 0.4, and 0.5 mg/mL were prepared in 0.6 M PBS (pH 7.0). For each concentration, 4 mL of solution was mixed with 20 μL of 15 mM ANS-Na in PBS, vortexed, and incubated in the dark at 25 ± 1 °C for 20 min. Then, 200 μL of the mixture was loaded into a black 96-well microplate, and fluorescence was measured (excitation 375 nm; emission 420–600 nm, 5 nm interval) using a multifunctional microplate reader.

The surface hydrophobicity index (H_0_) was calculated as the slope of the linear regression between the maximum fluorescence intensity (typically at 470–480 nm) and protein concentration. All measurements were performed in triplicate, and the ANS solution was freshly prepared.

### 2.5 Characterization of HIPEs

#### 2.5.1 Visual appearance

The visual appearance of HIPEs stabilized by MP and various MP-TA conjugates was recorded using a camera (Huawei, China) under consistent lighting conditions.

#### 2.5.2 Confocal laser scanning microscopy (CLSM)

The microstructure of HIPEs stabilized by different MP-TA conjugates was observed using a CLSM (Leica TCS SP8 X, Heidelberg, Germany). Nile Red (0.1 % w/v in ethanol) and Nile Blue (0.1 % w/v in deionized water) stock solutions were freshly prepared before use. Specifically, 1 mL of each HIPE sample was mixed with 40 μL each of Nile Red and Nile Blue dye solutions. After vortexed, the mixtures were incubated in the dark at 25 °C for 3 h. A drop of the stained emulsion was then placed on a microscope slide and observed using a 40 × objective lens. Excitation wavelengths for Nile Red and Nile Blue were set at 488 nm and 633 nm, respectively. Each sample was analyzed in triplicate to ensure data reliability.

#### 2.5.3 Droplet size distribution

The volume-weighted mean diameter (D_4_,_3_), surface-weighted mean diameters (D_3,2_) and droplet dispersion of the emulsions were determined using a Malvern Mastersizer 3000 (Malvern Instruments Ltd., England). HIPEs were appropriately diluted with ultrapure water prior to measurement, with an obscuration level of 10–15 %. Each measurement was performed five times per sample for 60 s each. The instrument automatically calculated the D_4_,_3_ value and the corresponding droplet size distribution.

#### 2.5.4 Rheology of MP/TA-stabilized HIPEs

##### 2.5.4.1 Shear viscosity

The shear viscosity of different HIPEs was measured using a rotational rheometer (Physica MCR 302e, Anton Paar, Austria). A parallel plate geometry (50 mm diameter, 1 mm gap) was used. At 25 °C under isothermal conditions, the shear rate was logarithmically increased from 0.1 s^−1^ to 1000 s^−1^, and the apparent viscosity was recorded accordingly. Each sample was measured at least three times. Between measurements, the rheometer system was cleaned with ultrapure water to remove residual sample.

##### 2.5.4.2 Strain sweep

The linear viscoelastic region (LVR) was determined through strain sweep tests. During the measurement, the frequency was fixed at 1.0 Hz, and the strain amplitude was logarithmically increased from 0.001 % to 10 %. By monitoring the response curves of the storage modulus (G′) and loss modulus (G″) as a function of strain, the strain range where modulus variation remained within ± 5 % was defined as the LVR. Samples were equilibrated for 5 min prior to testing, and each test was repeated in triplicate.

##### 2.5.4.3 Yield stress

Yield stress was evaluated using a stress sweep test. Specifically, the applied stress was logarithmically increased from 1 Pa to 1000 Pa, and the apparent viscosity (η) was recorded as a function of shear stress (σ). Rheoplus/32 software was used to analyze η–σ curves by second-derivative processing. The stress corresponding to a 5 % increase in viscosity was defined as the yield stress.

##### 2.5.4.4 Frequency sweep

Rheological behavior was further evaluated using frequency sweep tests within the previously determined LVR (strain amplitude: 0.01 %). Specifically, the angular frequency (ω) was logarithmically increased from 0.1 to 100 rad/s, and G′ and G″ were recorded as functions of ω. The test was conducted at 25 ± 0.1 °C with Peltier temperature control using parallel plates (gap width: 1 mm). Samples were equilibrated for 2 min before testing, and all measurements were performed in triplicate. To describe the relationship between the complex modulus (G*) and frequency, the weak gel model proposed by Gabriele et al. [20] was applied:

(7) $${{G^{*}\left(\omega \right)=A}_{f}\times \omega }^{\frac{1}{z}}$$

where A_f_ is the gel strength coefficient and z is the structural parameter, with higher z values indicating greater network connectivity.

#### 2.5.5 Stability of HIPEs

##### 2.5.5.1 Storage stability

The storage stability of the emulsion systems was assessed using confocal laser scanning microscopy (CLSM). Freshly prepared emulsions were stored at 4 ± 1 °C for 14 days. After the storage period, 1 mL of each sample was collected, stained, and imaged as described in Section 2.5.2. By comparing the microstructures of the stored and fresh samples (day 0), changes in droplet size distribution and signs of droplet aggregation or coalescence were evaluated.

##### 2.5.5.2 Heating stability

The heating stability of the emulsions was evaluated using optical microscopy. Specifically, 5 mL of freshly prepared emulsion was transferred to a 10 mL centrifuge tube and heated via steam treatment for 1 h. Immediately after heating, the samples were cooled in an ice bath to 25 °C. Before and after heating, a small amount of sample was placed between a microscope slide and coverslip. Bright-field images were captured at 40 × magnification. For each sample, at least five random fields of view were selected for observation.

##### 2.5.5.3 Centrifugal stability

Freshly prepared emulsions (16 mL) was transferred into a 50 mL centrifuge tube. Centrifugation was performed at 15,000 × g for 10 min using a high-speed refrigerated centrifuge (Thermo Scientific Sorvall ST 16R), with temperature maintained at 4 °C. Immediately after centrifugation, the tube was placed upright, and the visual appearance of the emulsion was photographed.

#### 2.5.6 3D printing of HIPEs

HIPEs were printed using a 3D food printer (Foodbot, China). A plastic nozzle with an inner diameter of 0.84 mm was used, and the printing speed was maintained at 15 mm/s. The first layer height was fixed at 0.84 mm, and a rectilinear infill pattern with 80 % infill density was used. To optimize the printing parameters, a 3 × 3 orthogonal design was conducted using a representative formulation (10 μM/g TA, 240 W HS), with a 15 × 15 × 10 mm cuboid serving as the test printing model.

#### 2.5.7 Statistical analysis

All experiments were performed in triplicate (n = 3); data are expressed as mean ± SD. Differences among groups were assessed by one-way ANOVA in IBM SPSS Statistics 26, with p < 0.05 considered significant. Plotting and curve fitting were carried out in OriginPro 2025.

## 3 Results and discussion

### 3.1 Characterization of MP-TA conjugates

#### 3.1.1 Visual appearance and surface morphology characterized by AFM

Ultrasound treatment markedly improved the transparency of the MP-TA conjugates, likely due to increased MP solubility (Fig. 1a). Upon addition of TA, the solution turned pale yellow, with increasing TA concentrations resulting in deeper coloration. These visible changes indicate polyphenol oxidation and progressive formation of covalent MP–TA conjugates. AFM was used to visualize the surface morphology of native MP and its conjugates in both 2D and 3D images (Fig. 1b). In the Control and TA-0 groups, ellipsoid-like filaments typical of MP were observed. Following TA addition, more compact MP-TA conjugates formed due to enhanced protein–polyphenol interactions. With increasing TA levels, progressively larger and rougher aggregates were observed, suggesting stronger intermolecular associations. Ultrasound treatment promoted protein unfolding, enabling disordered aggregates to rearrange into more uniform structures [21]. As ultrasonic intensity increased, protein unfolding was further enhanced, strengthening interactions with TA and leading to the formation of taller aggregates, as confirmed by AFM images.

#### 3.1.2 Particle size and zeta potential

**Fig. 2:**
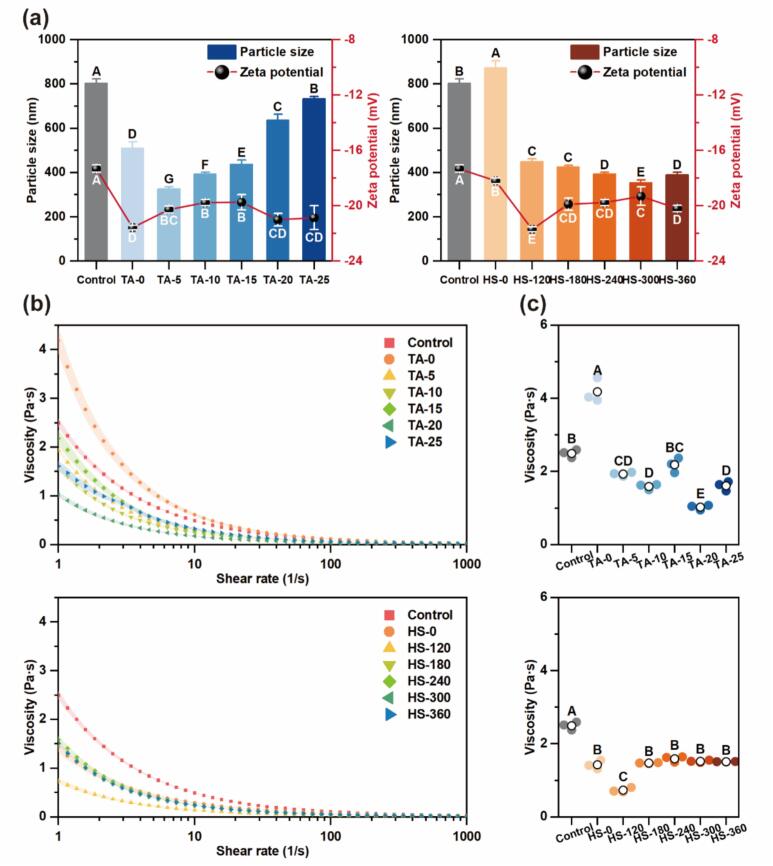
Particle size, zeta-potential (a), apparent viscosity (b) and zero-shear viscosity (c) of MP and different MP-TA conjugates. Different capital letters indicated significant differences (P < 0.05) between samples.

Particle size and surface charge of MP–TA conjugates prepared via HS-assisted covalent modification were analyzed by DLS (Fig. 2a). Crosslinking with TA alone increased the size of MP particles from 803.8 ± 19.5 nm to 872.4 ± 33.1 nm, consistent with polyphenol-induced aggregation, although excessive aggregation can impair colloidal stability [22]. Interestingly, with increasing HS intensity, particle size decreased progressively, likely due to cavitation, shear, and turbulence effects that disrupted large aggregates and improved dispersion uniformity [23]. When combined with TA, ultrasound further promoted the formation of more compact conjugates, possibly by altering secondary structures and stabilizing micellar architectures through protein bridging [24]. Nonetheless, with continuous TA addition, the particle size increased again due to extensive crosslinking between TA and MP chains, forming larger polymeric structures.

Zeta potential was measured to characterize particle surface charge. A higher absolute value of zeta potential generally reflects a stronger surface charge, which enhances electrostatic repulsion and prevents particle aggregation [25]. As illustrated in Fig. 2a, the native MP exhibited a zeta potential of −17.3 ± 0.26 mV, indicating a negatively charged surface. Covalent modification increased the absolute value, likely due to reactions between TA and MP amino groups, as supported by FTIR results in Section 3.1.4, and consistent with mechanisms such as Michael addition previously reported in protein–polyphenol systems. Moreover, ultrasound-assisted complexation may also redistribute surface charges via protein unfolding and conformational rearrangement.

#### 3.1.3 Shear viscosity

The shear viscosity profile provides valuable insight into the physicochemical properties of proteins and the strength of intermolecular interactions. As shown in Fig. 2b, all samples exhibited shear-thinning behavior, with a sharp viscosity decrease at low shear rates followed by a gradual approach to an infinite-shear plateau. This non-Newtonian behavior likely results from the partial disruption of protein networks under shear stress [26]. At the onset of shearing, polymer chains behave as tangled, flexible coils; while individual chains attempt to move and rearrange, chain entanglements help maintain structural integrity. Zero-shear viscosity (Fig. 2c), determined under low-shear conditions, provides further information on the internal network structure. All MP–TA conjugates exhibited lower zero-shear viscosity than native MP. This reduction may result from the decreased particle size, which reduces flow resistance, and the increased absolute zeta potential, which may weaken interparticle entanglements and attractive forces, thereby enhancing dispersion fluidity.

#### 3.1.4 FTIR and secondary structure

**Fig. 3:**
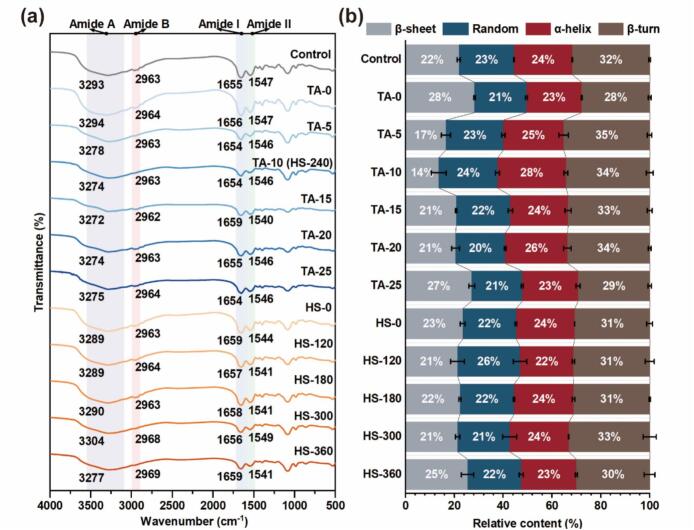
FTIR spectrum (a) and secondary structure relative content (b) of MP and different MP-TA conjugates.

FTIR spectroscopy was used to examine covalent interactions between MP and TA under ultrasound-assisted conditions. In native MP (Fig. 3a), characteristic peaks were located at 3293 cm^−1^ (amide A, N–H/O–H stretching), 2963 cm^−1^ (amide B, N–H/O–H stretching and C–H symmetric stretching), 1655 cm^−1^ (amide I, C=O stretching), and 1547 cm^−1^ (amide II, C–N/N–H bending) [27,28]. As shown in Fig. 3a, with increasing ultrasound intensity, progressively larger shifts were observed in the amide A and amide I bands of MP–TA conjugates compared with native MP, indicating ultrasound-enhanced structural modifications. These shifts suggest that ultrasound promoted reactions between TA phenolic –OH and MP amino groups under alkaline conditions [29]. For example, in TA-15 groups, the amide I peak shifted from 1655 to 1659 cm^−1^, consistent with altered C=O stretching, while the amide II peak moved from 1547 to 1540 cm^−1^, possibly due to Michael addition or Schiff base formation, as reported in similar protein–polyphenol systems [30]. These findings confirm multiple covalent linkages at the MP–TA interface and highlight the role of ultrasound in strengthening interfacial associations.

Secondary structure changes were analyzed via Gaussian fitting of the amide I band (1600–1700 cm^−1^), where α-helix, β-sheet, random coil, and β-turn components were identified within standard wavenumber ranges [18]. Fig. 3b shows that native MP contained 24 % α-helix, 22 % β-sheet, 32 % random coil, and 23 % β-turn. After ultrasound treatment, β-sheet content increased significantly, while α-helix decreased, likely due to disruption of intramolecular hydrogen bonding by cavitation-induced shear and heat [31].

TA conjugation led to non-linear changes in β-sheet and random coil content: at low TA levels, hydrogen bond disruption decreased β-sheet content, while at higher concentrations, π–π stacking and hydrophobic interactions facilitated reorganization [32]. α-Helix content mildly increased, peaking at 28 % in the TA-10 group, suggesting that TA binding contributes to structural stabilization [33]. Similar findings were reported by Hu et al. [34]. For HS groups, high ultrasound had a more pronounced effect. With HS intensity in 120 W, random coil content initially increased while α-helix decreased, indicating enhanced structural disorder. At intensities > 180 W, a partial recovery in α-helix content occurred, suggesting that polyphenol rigid domains may limit excessive unfolding. At 300 W, β-sheet content sharply increased, reflecting extensive unfolding with flexible and open protein conformations. These results align with previous studies on ultrasound-induced secondary structure transitions in soy proteins [35].

#### 3.1.5 Tertiary structure

##### 3.1.5.1 Intrinsic fluorescence spectroscopy

**Fig. 4:**
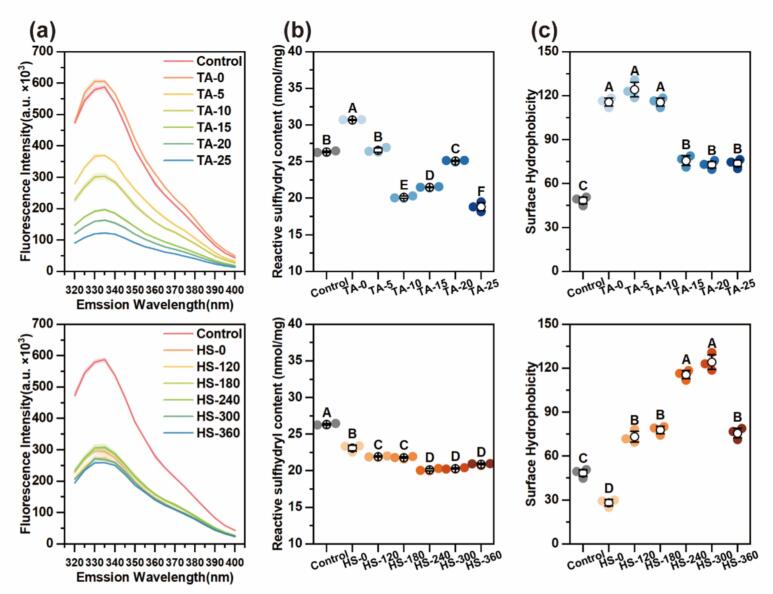
Intrinsic fluorescence spectra (a), reactive sulfhydryl content (b) and surface hydrophobicity (c) of MP and different MP-TA conjugates. Different capital letters indicated significant differences (P < 0.05) between samples.

Intrinsic fluorescence spectroscopy was employed to evaluate conformational changes in MP upon covalent modification. The fluorescence emission of proteins primarily originates from tryptophan (Trp) residues, which are highly sensitive to their surrounding polarity. In native MP, Trp residues are typically buried within hydrophobic cores. Upon protein unfolding, these residues become exposed to aqueous environments, resulting in fluorescence quenching [36]. As shown in Fig. 4a, all MP-TA conjugates exhibited a notable decrease in fluorescence intensity compared to the control. This quenching effect intensified with increasing TA concentration. Such behavior suggests progressive protein unfolding and exposure of Trp residues to more polar surroundings, driven by TA binding and ultrasonic disruption. A redshift in the maximum emission wavelength further confirmed the enhanced polarity around Trp residues, consistent with previous reports [37]. In HS groups, a further decline in fluorescence intensity was observed with increasing ultrasonic power. This enhanced quenching effect likely arises from greater protein unfolding, which exposes more binding sites for TA, thereby amplifying the interaction and fluorescence suppression. Similar synergistic effects between ultrasonic treatment and polyphenol conjugation have been observed in soy protein systems [38].

Overall, these findings indicate that TA-mediated covalent modification, particularly under ultrasound assistance, induces partial unfolding and conformational extension of MP. The associated fluorescence quenching reflects enhanced exposure of Trp residues and supports the occurrence of strong MP–TA interactions at the molecular level.

##### 3.1.5.2 Reactive sulfhydryl (R-SH) content

Reactive sulfhydryl (−SH) groups are critical for maintaining protein tertiary structure and their high chemical reactivity enables interactions with polyphenols, thereby influencing protein conformation and stability [39]. As shown in Fig. 4b, the native MP exhibited a free sulfhydryl content of 26.33 ± 0.10 mmol/mg. Ultrasound treatment alone (TA-0 group) significantly increased R–SH content, suggesting enhanced exposure of buried thiol groups or disruption of disulfide bonds. This finding highlights the structural loosening effect of ultrasound. However, upon TA conjugation, the R–SH content decreased progressively. This reduction is likely due to the covalent interaction between protein thiol groups and TA hydroxyl groups, which either consume –SH directly or promote disulfide bond (−S-S-) formation [40]. The decline in sulfhydryl content was further intensified under ultrasound-assisted conditions, indicating that ultrasound facilitates molecular contact and accelerates conjugation efficiency. Similar reductions in free –SH content have been reported for other protein–polyphenol systems, such as ovalbumin–EGCG conjugates [41], Overall, these results confirm that both ultrasound and TA play synergistic roles in modifying MP tertiary structure by regulating sulfhydryl group availability.

##### 3.1.5.3 Surface hydrophobicity

Surface hydrophobicity is a critical determinant of protein functionality, closely linked to conformational state and interfacial behavior. The ANS fluorescence probe was used to evaluate the exposure of hydrophobic residues in native MP and MP–TA conjugates (Fig. 4c). Compared to native MP, the TA-0 group exhibited a significant increase in surface hydrophobicity, indicating that ultrasound treatment enhanced the exposure of buried hydrophobic domains. This effect is attributed to acoustic cavitation, which disrupts tertiary structure and relocates hydrophobic amino acids to the surface [42]. These structural changes are consistent with secondary structure transitions discussed in Section 3.1.4. With increasing TA concentration, surface hydrophobicity progressively decreased. This trend likely results from polyphenol-induced crosslinking and aggregation, which could shield hydrophobic sites and reduce ANS binding. The results aligned well with the particle size changes reported for the TA-treated samples (Fig. 2a). Notably, a sharp decline was observed at TA-15, suggesting that TA-10 may represent an inflection point for maximal hydrophobic exposure. Similar critical thresholds have been reported for MP-genipin systems [17].

In HS groups, increasing HS intensity (120–300 W) led to elevated surface hydrophobicity. This enhancement reflects both fragmentation of protein aggregates and unfolding-induced exposure of internal hydrophobic groups [43]. However, at the highest intensity (360 W), surface hydrophobicity declined, possibly due to reaggregation or partial refolding of protein structures, which reburies hydrophobic domains.

These results indicate that ultrasound and TA synergistically modulate protein conformation by exposing or masking hydrophobic groups. The structural transitions observed here approximately align with concurrent changes in particle size, morphology, and structural features, confirming substantial rearrangement of MP conformation upon conjugation.

#### 3.1.6 Interfacial mechanics: Tension and modulus

**Fig. 5:**
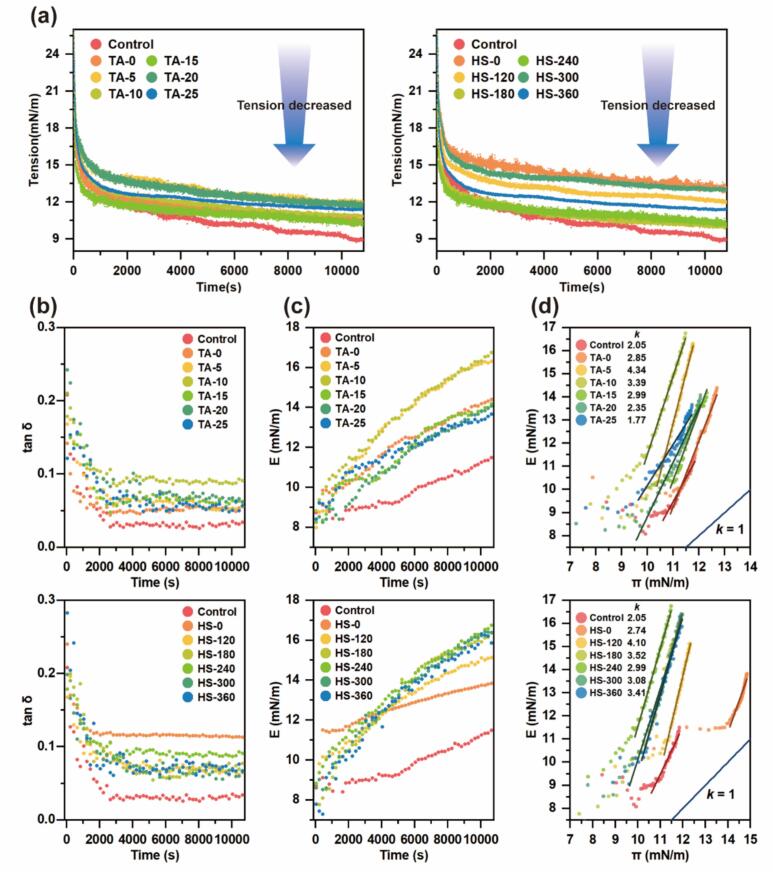
The interfacial adsorption analysis and dilatational rheological properties of MP and different MP-TA conjugates: the interfacial tension (a), the time dependence of tan δ (b), the time dependence of dilatational modulus (c) and the interfacial pressure (π) dependence of dilatational modulus (d), the line whose slope K = 1 represents the characteristic behaviour of ideal gas.

Interfacial mechanics govern emulsion formation and stability, therefore, the dynamic interfacial tension and dilatational modulus of MP and MP-TA conjugates during adsorption at the oil–water interface was monitored (Fig. 5).

As shown in Fig. 5a, all samples exhibited a rapid decrease in interfacial tension within ∼ 1000 s, followed by a gradual approach to a plateau. The initial drop reflects the diffusion‑controlled adsorption of protein species to the interface, which increases the surface excess (Γ) and surface pressure (π), thereby lowering the interfacial tension. As adsorption proceeds, lateral crowding and electrostatic repulsion slow further adsorption; concurrently, interfacial rearrangement occurs in which myosin/actin domains in MP re‑orient (hydrophobic heads toward oil, hydrophilic tails toward water), tightening the interfacial layer [44]. The subsequent plateau represents a dynamic adsorption–desorption/rearrangement steady state (i.e., θ and Γ become quasi‑constant under these conditions), rather than cessation of molecular motion [44]. This two‑stage kinetic profile (fast diffusion/adsorption → slower rearrangement/consolidation) is characteristic of protein‑stabilized interfaces and was also observed here for MP-TA conjugates, albeit with treatment‑dependent magnitudes. Comparable two‑stage profiles are widely reported for protein‑based interfaces [45,46].

Equilibrium interfacial tension is commonly used to assess the emulsifying capacity of compounds, with lower values indicating better interfacial stabilization due to a favorable balance between surface charge and hydrophobicity [47]. In this study, although all samples effectively reduced interfacial tension, native MP exhibited the lowest equilibrium value. As indicated by our previous results (Fig. 3, Fig. 4), ultrasound-assisted conjugation with TA induced unfolding of MP and increased its surface hydrophobicity. This structural relaxation promotes the formation of hydrophobic domains that more readily anchor at the interface, thus enhancing adsorption efficiency. For instance, Karefyllakis et al. [48] reported enhanced surface activity and reduced interfacial tension for sunflower protein-chlorogenic acid conjugates.

Interestingly, the MP-TA conjugates showed increased equilibrium interfacial tension, contrary to previous reports. This unexpected trend may be explained by steric hindrance at the interface. Apart from surface hydrophobicity, particle crowding—also referred to as steric exclusion—can limit adsorption and affect tension reduction [49]. TA-induced covalent crosslinking increased the absolute zeta potential of MP-TA conjugates (Fig. 2a), potentially intensifying electrostatic and spatial repulsion, which in turn hindered interfacial adsorption. Similarly, Sausse et al. [50] found that polyphenols could introduce energy barriers impeding protein adsorption. In contrast, Wei et al. [51] reported that protein–EGCG conjugates enhanced interfacial activity and antioxidant capacity. These conflicting findings suggest that interfacial behavior is governed by a complex interplay of molecular structure, surface charge, and environmental conditions. Nevertheless, despite the slightly elevated equilibrium tension, MP-TA conjugates still substantially lowered interfacial tension, supporting their potential as emulsifiers.

In addition to interfacial adsorption, the rheological properties of protein films are critical to emulsion stability. Dilatational interfacial rheology characterizes the film’s mechanical strength and its resistance to deformation. Upon adsorption, protein molecules undergo mutual compression, decreasing the interfacial area per molecule and resulting in the development of the dilatational modulus (E), which includes both elastic (E_d_) and viscous (E_v_) components [52].

The time-dependent evolution of the phase angle tangent (tanδ = E_v_/E_d_) is illustrated in Fig. 5b. All samples displayed a rapid initial decrease in tanδ, reaching a stable value between 0 and 1 by 10,800 s. This behavior indicates that elasticity predominated over viscosity (E_d_ ≫ E_v_), reflecting the formation of primarily elastic interfacial films [19]. Compared to native MP, all modified samples exhibited higher equilibrium tanδ values, implying increased film flexibility following covalent or ultrasound-assisted treatment.

As shown in Fig. 5c, all samples exhibited a rapid increase in E during the early adsorption phase, consistent with the initial decline in interfacial tension (Fig. 5a), indicating diffusion-controlled film formation [53]. Although high-intensity ultrasound had a limited effect on E, increasing TA concentration enhanced E up to 10 μM/g, beyond which a sharp decline was observed—likely due to reduced surface hydrophobicity at higher TA levels (Fig. 4c). All MP–TA conjugates exhibited higher E values than native MP, suggesting the formation of mechanically more robust interfacial films. This enhancement is attributed to increased packing density and strengthened intermolecular interactions [46]. In fact, thin but tightly packed globular protein films can be brittle upon deformation [54]. The formation of viscoelastic particle networks at the interface may also contribute to the elevated modulus, as supported by previous findings on protein–polysaccharide systems [55].

E increased with interfacial pressure (π) for all samples, as shown in Fig. 5d, indicating enhanced intermolecular interactions at the interface [56]. Theoretically, ideal gas-like adsorption yields an E-π slope of 1 [57]; however, all curves showed higher slopes, suggesting contributions from protein–protein interactions and molecular packing [58]. The control group exhibited the lowest slope, likely due to weaker cohesion and insufficient steric stabilization [59]. In contrast, MP-TA conjugates displayed a concentration-dependent increase in slope, peaking at moderate TA levels, consistent with the E results in Fig. 5c. A similar trend was observed in HS groups, further confirming that ultrasound enhances MP-TA interactions and interfacial film integrity.

In summary, interfacial tension data (Fig. 5a) indicate that MP-TA conjugates exhibit effective interfacial adsorption and emulsifying ability. Moreover, interfacial dilatational analysis (Fig. 5 b–d) shows that these conjugates form viscoelastic films with enhanced mechanical strength and moderate flexibility, which contribute to greater resistance against deformation.

### 3.2 Characterization of HIPEs stabilized by MP-TA conjugate

#### 3.2.1 Visual appearance and micromorphology of HIPEs

**Fig. 6:**
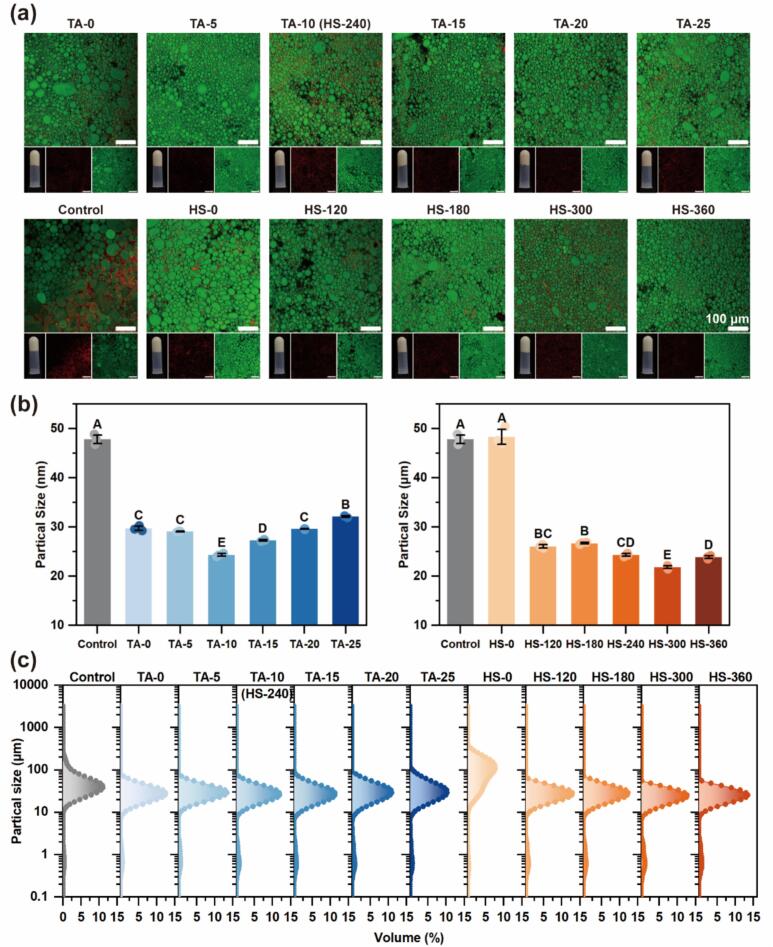
Visual appearance, CLSM images (a), droplet size D_4,3_ (b) and distribution (c) of HIPEs stabilized by MP and different MP-TA conjugates. Different capital letters indicated significant differences (P < 0.05) between samples. (The scale bars are 100 μm in CLSM images.).

The visual appearance and CLSM images of HIPEs are shown in Fig. 6a. All emulsions remained inverted without flowing, confirming successful HIPE formation in all groups. CLSM revealed closely packed droplets—a typical HIPE feature—indicating high internal phase packing. Notably, HIPEs stabilized by MP-TA conjugates prepared under ultrasound (≥180  W) and moderate TA concentrations (<25  μM/g) exhibited smaller, more uniformly distributed droplets. This structural refinement suppressed coalescence and enhanced emulsion integrity. The improved stability likely arises from reduced droplet size, enhanced Brownian motion [59], and the formation of stronger interfacial films, consistent with the dilatational modulus data (Fig. 5c). In this regime, ultrasound and MP–TA interactions acted synergistically to stabilize droplets during emulsification and storage, as also observed in cinnamaldehyde-TA nanoemulsions [60]. In contrast, excessive TA induced the formation of large MP-TA aggregates, limiting interfacial adsorption and causing unabsorbed conjugates to accumulate in the continuous phase. This led to visible aggregation of emulsion droplets and a consequent loss of HIPE structural integrity.

#### 3.2.2 Droplet size and distribution of HIPEs

Droplet size plays a critical role in determining emulsion stability, with smaller droplets typically enhancing resistance to flocculation and phase separation [61]. Fig. 6, Fig. 6 show the volume-weighted mean diameter (D_4_,_3_) and size distribution of HIPEs stabilized by MP and MP-TA conjugates. Ultrasound-assisted covalent conjugation markedly reduced droplet size and improved size uniformity, resulting in more compact emulsions. The HS-0 group, lacking ultrasound during conjugation, exhibited the broadest size distribution, indicating inefficient particle dispersion, which may be linked to its relatively high equilibrium interfacial tension (Fig. 5a). Although TA improved interfacial film formation, the absence of ultrasound limited particle unfolding and interfacial adsorption efficiency. The smallest droplet size and narrowest distribution were achieved at 10 μM/g TA and 300 W ultrasound, confirming these as optimal conditions for enhanced physical stability. This trend corresponds with prior structural data (Section 3.1.2), where ultrasound facilitated MP unfolding and TA binding. In contrast, 360 W ultrasound led to the formation of insoluble aggregates, impairing emulsification. Additionally, the surface-weighted mean diameter (D_3_,_2_) (Fig. S2) showed a similar decreasing trend, further supporting the conclusions drawn from D_4_,_3_. Furthermore, these results are further supported by the interfacial mechanics discussed in Section 3.1.6, which showed that stronger interfacial protein networks improved film elasticity and resistance to coalescence, ultimately contributing to smaller and more stable droplets.

#### 3.2.3 Rheology of HIPEs

**Fig. 7:**
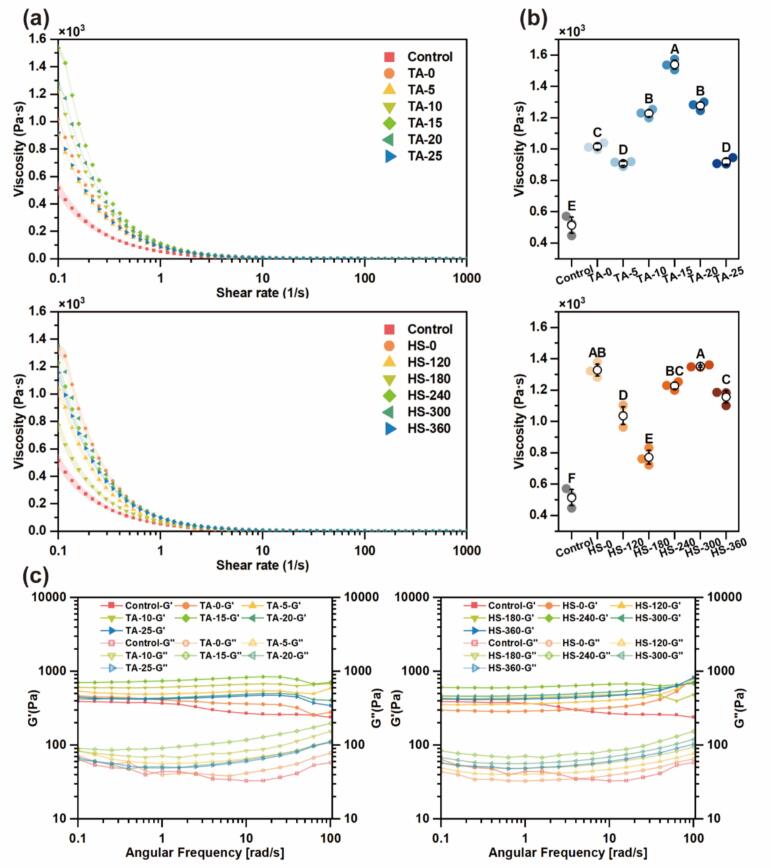
Shear rate sweep (a), zero-shear viscosity (b) and frequency sweep (c) of HIPEs stabilized by MP and different MP-TA conjugates. Different capital letters indicated significant differences (P < 0.05) between samples.

Rheological analysis characterizes the deformation and flow behavior of HIPEs, which is critical for assessing their mechanical performance and application potential. As shown in Fig. 7a, all samples exhibited shear-thinning behavior typical of pseudoplastic fluids, with apparent viscosity decreasing as shear rate increased (0.1–1000 s^−1^). This response reflects the disruption of internal gel-like networks under shear [62]. Fig. 7b shows that zero-shear viscosity was elevated in all treated samples compared to the control, indicating increased resistance to flow. Notably, viscosity increased with TA concentration, peaking at intermediate levels, likely due to reduced droplet size and enhanced droplet packing (Section 3.2.2; [16]). However, at concentrations above 20 μM/g, viscosity declined, potentially due to larger aggregates (Fig. 2a) introducing steric hindrance and repulsive forces that compromised the oil–water network structure [63].

Frequency sweep tests were used to assess the dynamic rheological behavior of HIPEs (Fig. 7c). G′ represents stored energy, while G″ reflects viscous dissipation [64]. In all cases, G′ exceeded G″ across the frequency range, demonstrating weak gel-like elasticity. The observed elasticity likely arises from tight droplet packing and the formation of MP-TA networks at both the interface and in the continuous phase [65].

The weak gel model was applied to complex modulus (G*) to evaluate structural integrity, with A_F_ and z representing interaction strength and flow unit number, respectively. Model fitting was satisfactory (R^2^ > 0.9). As shown in Table 1, both parameters initially increased and then declined with rising ultrasound intensity or TA concentration, peaking at 240 W and 15 μM/g. These results suggest that moderate treatment promotes denser, more ordered networks, enhancing emulsion resilience. In contrast, excessive TA addition may lead to large MP-TA aggregates (Fig. 2a), which impair interfacial adsorption and film strength—consistent with interfacial findings in Section 3.1.6 [66].

**Table 1:** The weak gel model parameters of HIPEs stabilized by MP and different MP-TA conjugates.

SN	Parameters of the weak gel		
	A_F_ (Pa⋅s ^1/z^)	z	R^2^
Control	369.8 ± 4.43	−7.83 ± 0.54	0.97
TA-0	414.03 ± 2.26	−18.9 ± 1.32	0.97
TA-5	499.46 ± 1.61	26.47 ± 1.39	0.98
TA-10 (HS-240)	618.19 ± 1.03	26.12 ± 0.7	0.99
TA-15	751.55 ± 1.82	20.3 ± 0.61	0.99
TA-20	440.87 ± 1.32	20.29 ± 0.75	0.99
TA-25	428.11 ± 1.23	23.97 ± 1.01	0.99
HS-0	290.96 ± 2.46	20.01 ± 2.06	0.93
HS-120	365.62 ± 2.29	20.61 ± 1.62	0.96
HS-180	442.94 ± 0.92	24.67 ± 0.78	0.99
HS-300	474.5 ± 2.59	20.4 ± 1.39	0.97
HS-360	425.07 ± 2.78	21.16 ± 1.79	0.95

**Fig. 8:**
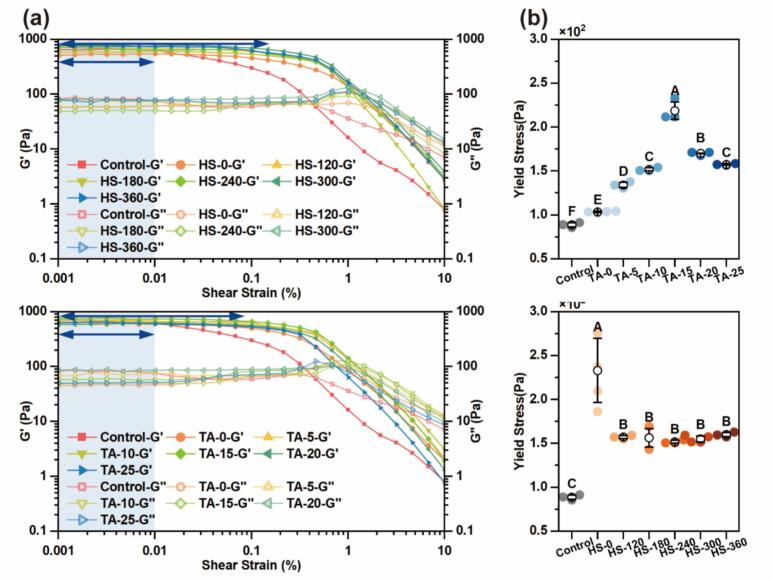
Strain sweep (a) and yield stress (b) of HIPEs stabilized by MP and different MP-TA conjugates. Different capital letters indicated significant differences (P < 0.05) between samples.

To further investigate the structural response of HIPEs under shear deformation, strain sweep and yield stress tests were conducted. Compared to HIPEs stabilized by native MP, MP-TA-stabilized HIPEs exhibited a broader LVR, indicating enhanced structural integrity under shear deformation (Fig. 8a). At strains below 0.01 %, G′ remained at least one order of magnitude greater than G″, confirming strong elastic dominance. This behavior reflects a robust internal network likely strengthened by MP-TA interactions. As strain increased, G′ declined while G″ slightly rose before intersecting, displaying weak overshoot behavior characteristic of structured emulsions with transient resistance to deformation [67].

Yield stress, which reflects the minimum force required to initiate flow, is a key indicator of HIPE structural robustness [68]. As shown in Fig. 8b, it increased with TA concentration, peaking at 15  μM/g, after which a decline was observed. This suggests that excessive TA addition disrupts droplet architecture, inducing a shift toward viscous-dominated behavior—consistent with the earlier frequency sweep results (Fig. 7c). Nonetheless, all MP-TA-stabilized HIPEs exhibited significantly higher yield stress than the control, likely due to the combined effects of reduced droplet size, enhanced shear elasticity, and the formation of a more rigid interfacial network [67].

### 3.3 Stability of HIPEs stabilized by MP/TA conjugates

**Fig. 9:**
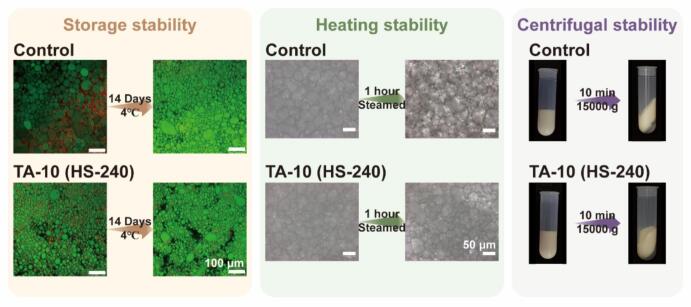
Stability of HIPEs stabilized by MP and different MP-TA conjugates. (The scale bars are 100 μm in CLSM images and 50 μm in OPM images, respectively.).

In food applications, emulsions are often exposed to mechanical and thermal stresses during processing, transport, and storage, making stability assessment a key criterion for functional evaluation [69]. Therefore, the storage, heating, and centrifugal stabilities of the HIPEs in this study were systematically evaluated (Figs. S3-S5). Fig. 9 further compares two representative formulations: HIPEs stabilized by native MP and by MP-TA conjugates prepared with 10 μM/g TA under 240 W ultrasound. After 14 days at 4 °C, the control sample exhibited pronounced droplet coalescence and flocculation, as observed by CLSM. In contrast, the TA-10 group largely retained its microstructure, showing only minor aggregation and markedly improved storage stability.

Upon heating at 100 °C for 60 min, all HIPEs displayed increased droplet sizes and structural heterogeneity, accompanied by droplet deformation. However, the TA-10 group exhibited significantly less enlargement than the control. Heat treatment promotes MP denaturation and crosslinking at the interface, reinforcing the emulsion matrix [70]. Despite moderate structural disruption, the TA-10 formulation retained a stable network, indicating improved thermal resilience conferred by MP-TA conjugates. Besides, under centrifugal stress (15,000 g, 10 min), the control HIPE showed visible oil separation and precipitates, likely due to sedimentation of unadsorbed protein aggregates. In contrast, all MP-TA-stabilized HIPEs exhibited no phase separation, suggesting that smaller droplet sizes and a reinforced interfacial network enhanced their resistance to centrifugal forces [18]. In summary, compared with native MP-stabilized HIPEs, those stabilized by MP-TA conjugates exhibited improved stability under long-term storage, heating, and centrifugal stress. These enhancements indicate the suitability of MP-TA conjugates for robust and food-grade emulsion applications.

### 3.4 3D printing of HIPEs ink

**Fig. 10:**
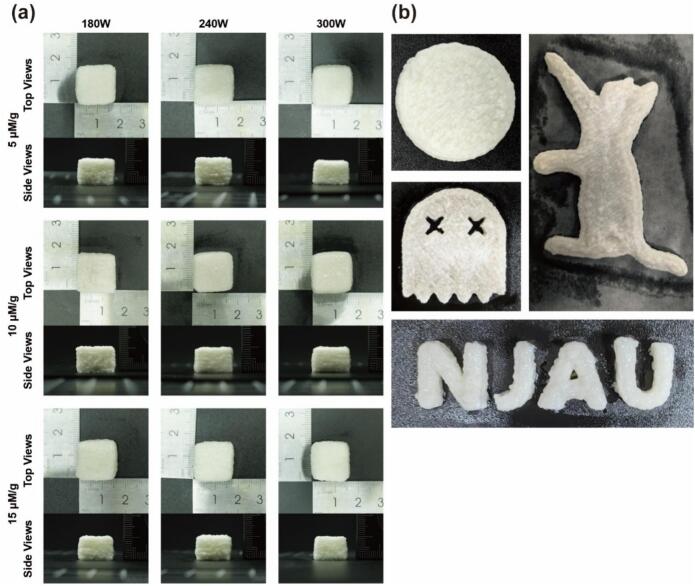
The visual appearance of 3D printed cuboid using HIPEs stabilized by different MP-TA conjugate (a). The visual appearance of 3D printed creature using HIPEs stabilized by 10 μM/g − 240 W MP-TA conjugate (b).

3D printing offers new possibilities for designing and fabricating customized food structures, and the performance of printable inks depends strongly on their rheological properties. Extrusion-based printing in particular requires sufficient structural integrity to retain the printed shape, as well as good extrudability [71]. Given the enhanced rheological behavior of HIPEs stabilized by MP-TA conjugates, their printability was further evaluated. A representative formulation (10  μM/g TA, 240  W HS) was selected to fabricate a 15 × 15 × 10  mm cuboid. A 3 × 3 orthogonal test was conducted to assess printing performance under varied conditions (Fig. 10a). All formulations demonstrated good shape retention and continuous filament extrusion. The 240 W-10  μM/g sample achieved the most precise geometry, with clear extrusion lines and minimal deformation. Additionally, this formulation enabled the successful printing of various complex geometries, including disc- and animal-shaped structures and letter patterns (Fig. 10b), highlighting the structural fidelity and versatility of MP-TA-stabilized HIPEs. Such systems may be suitable for both aesthetic and functional food applications, facilitating personalized design and novel product development.

## 4 Conclusion

The study demonstrated that ultrasound-assisted TA conjugation effectively modulated MP conformation, strengthened interfacial films, and enhanced their viscoelasticity (elevated dilatational moduli). These molecular and interfacial changes translated into HIPEs with smaller and more uniform droplets, higher viscosity, a broader linear viscoelastic region, and increased yield stress, conferring superior resistance to coalescence and oiling-off during storage, heating, and centrifugation. Among the investigated conditions, the formulation with 10μM g^−1^ TA and 240 W ultrasound provided the best overall balance of interfacial mechanics, emulsion stability, and 3D printability. Collectively, the results clarify how synergistic covalent conjugation and ultrasound govern MP interfacial assembly and translate to macroscopic performance, offering a practical route to engineer robust, protein-based HIPEs for food structuring. Further research focusing on gastrointestinal digestion and nutrient-delivery behavior of MP-TA-stabilized HIPEs would help elucidate how the modified interfacial structures influence bioaccessibility and release kinetics, thereby advancing their potential applications in functional and structured foods.

## CRediT authorship contribution statement

**Zitong Dong:** Conceptualization, Methodology, Validation, Formal analysis, Investigation, Data curation, Writing – original draft, Writing – review & editing, Visualization. **Feiyu Zhang:** Conceptualization, Investigation, Resources, Writing – review & editing, Visualization. **Peng Wang:** Conceptualization, Writing – review & editing, Resources. **Xinglian Xu:** Conceptualization, Project administration, Funding acquisition, Supervision, Resources, Writing – review & editing.

## Declaration of competing interest

The authors declare that they have no known competing financial interests or personal relationships that could have appeared to influence the work reported in this paper.
