# Supplementary material for: Ultrasound-assisted tannic acid crosslinking of myofibrillar protein-stabilized high internal phase emulsions: Enhanced stability, mechanical properties, and 3D printability
Source: Ultrason Sonochem. 2025 Dec 2;124:107703. doi: 10.1016/j.ultsonch.2025.107703 (PMC12743447; doi:10.1016/j.ultsonch.2025.107703)

**
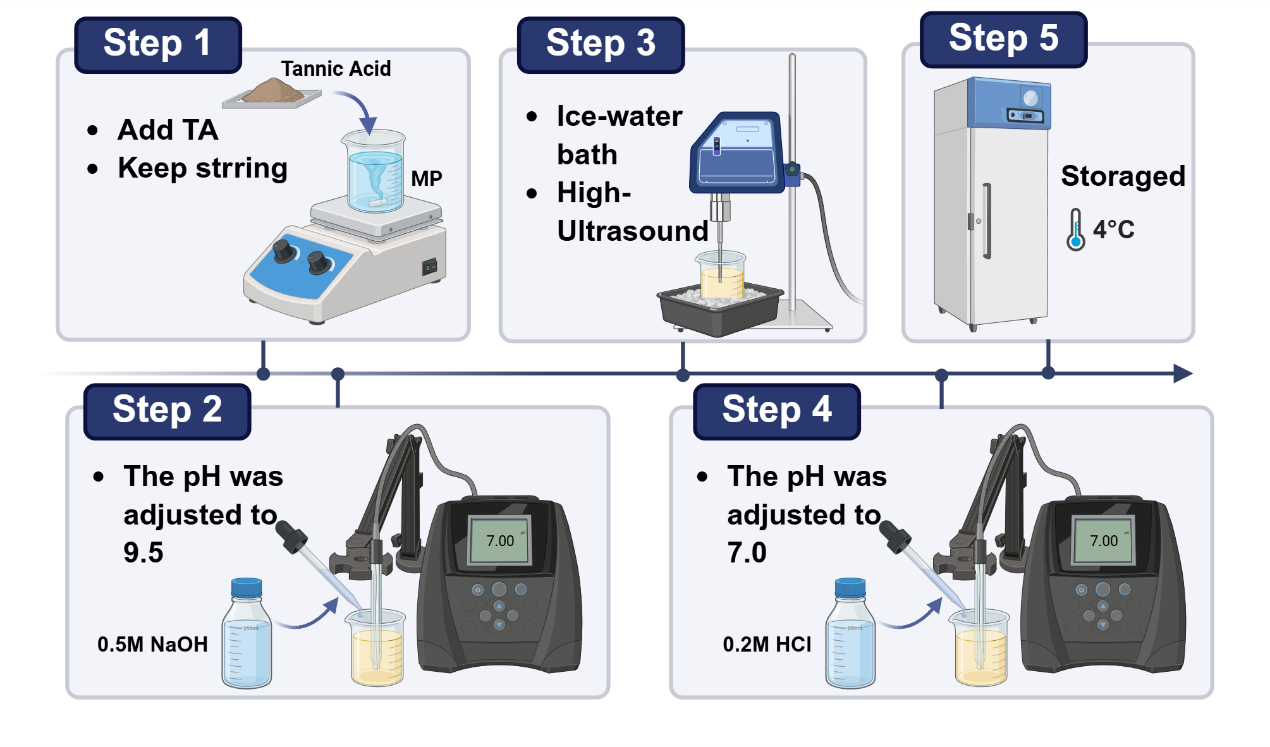
Fig.S1** The preparation method of MP-TA conjugates.

**Fig.S2** Surface-weighted mean diameters (D_3,2_) of HIPEs stabilized by MP and different **MP-TA conjugates**.
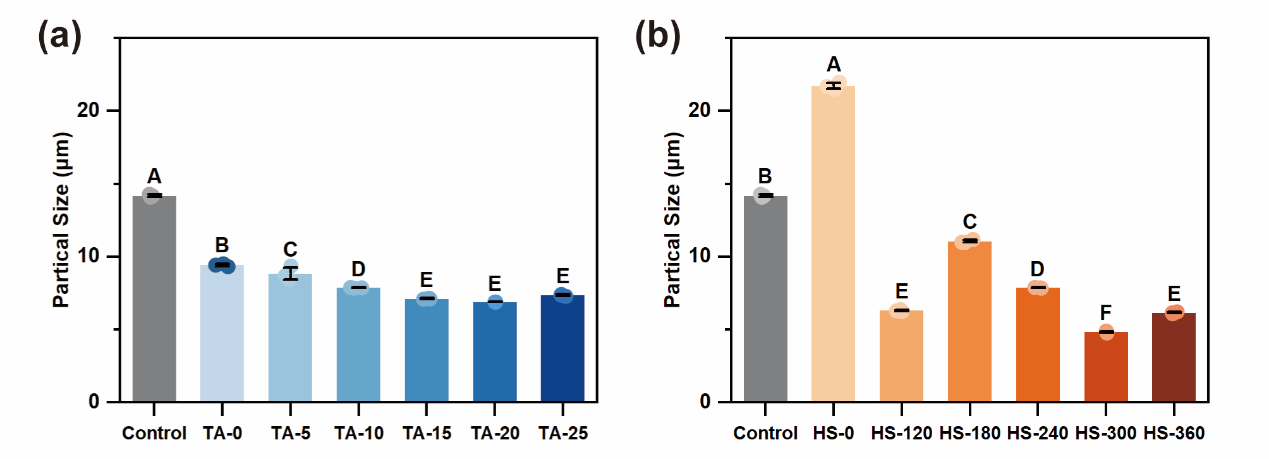


**
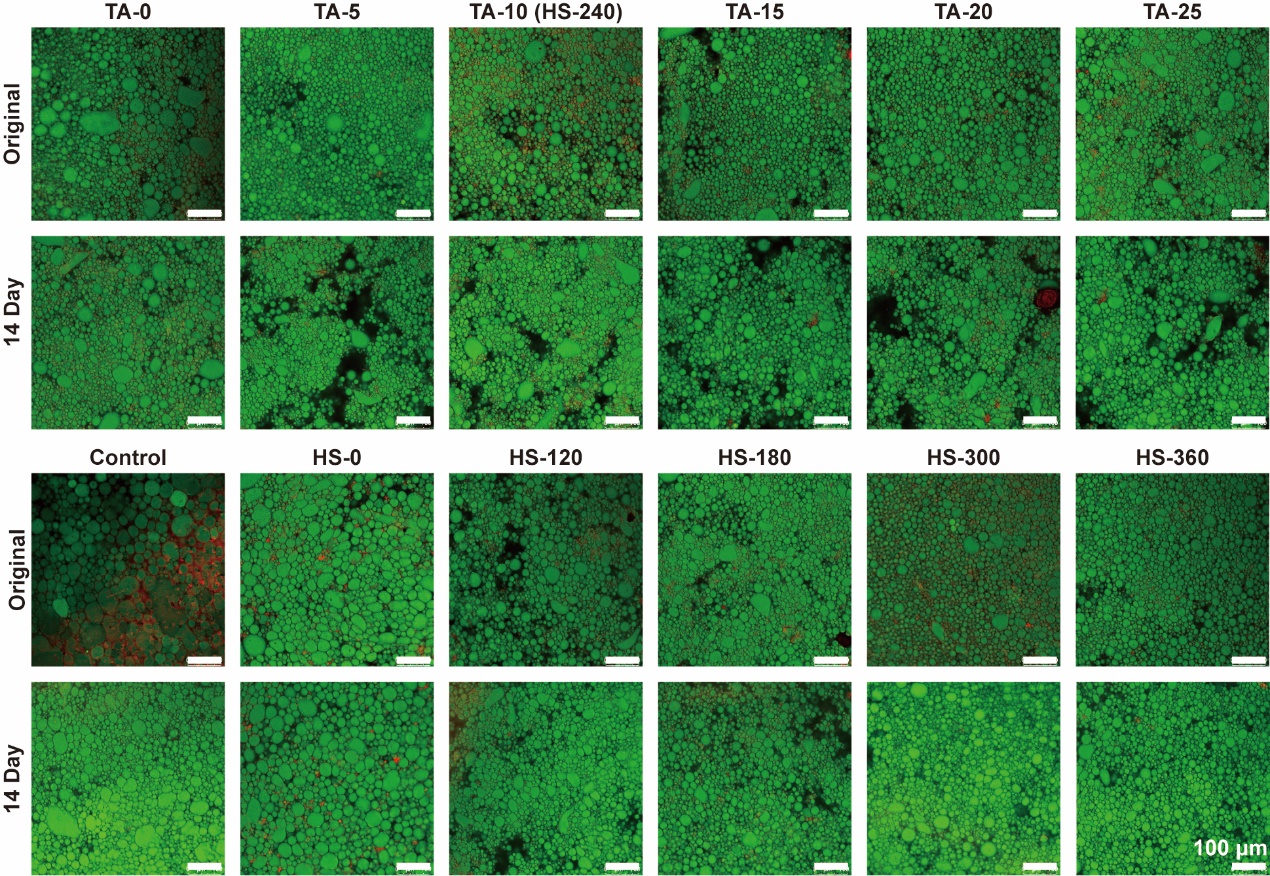
Fig.S3** The storage stability of HIPEs stabilized by MP and different **MP-TA conjugates**.

**
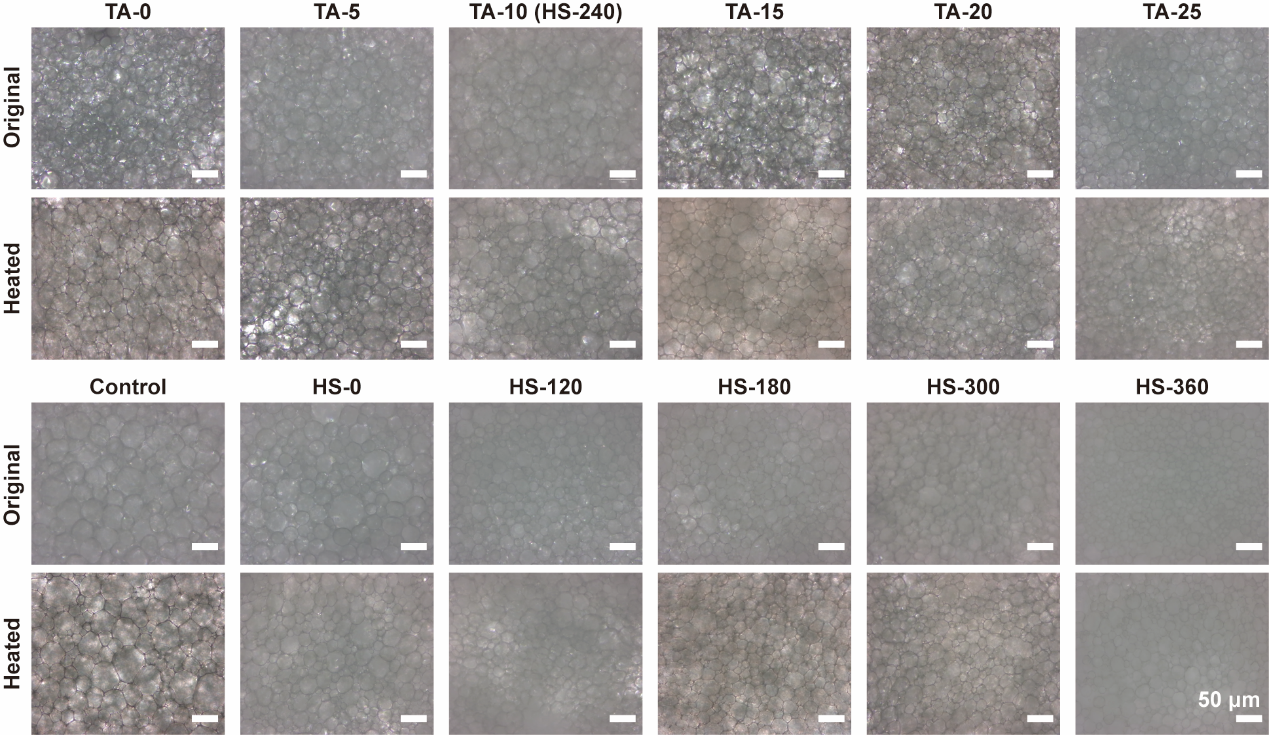
Fig. S4** The heating stability of HIPEs stabilized by MP and different **MP-TA conjugates**.

**Fig.S5** The centrifugal stability of HIPEs stabilized by MP and different **MP-TA conjugates**
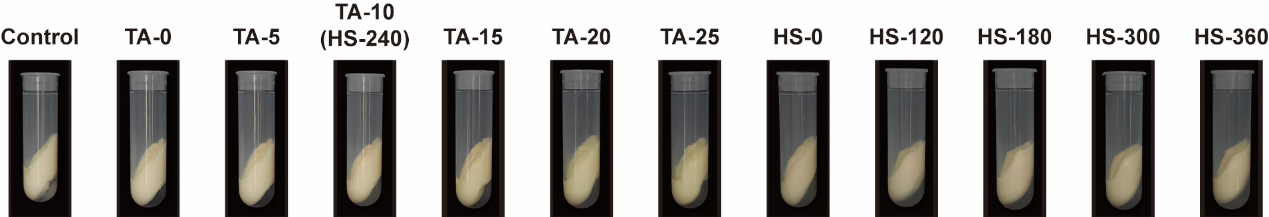

Supplement: Supplementary Data 1 [file mmc1.docx]
